# Association Between Smoking and SARS-CoV-2 Infection: Cross-sectional Study of the EPICOVID19 Internet-Based Survey

**DOI:** 10.2196/27091

**Published:** 2021-04-28

**Authors:** Federica Prinelli, Fabrizio Bianchi, Gaspare Drago, Silvia Ruggieri, Aleksandra Sojic, Nithiya Jesuthasan, Sabrina Molinaro, Luca Bastiani, Stefania Maggi, Marianna Noale, Massimo Galli, Andrea Giacomelli, Raffaele Antonelli Incalzi, Fulvio Adorni, Fabio Cibella, EPICOVID19 Working Group

**Affiliations:** 1 Institute of Biomedical Technologies National Research Council Segrate (MI) Italy; 2 Institute of Clinical Physiology National Research Council Pisa Italy; 3 Institute for Biomedical Research and Innovation National Research Council Palermo Italy; 4 Institute of Neuroscience National Research Council Padova Italy; 5 Infectious Diseases Unit, Department of Biomedical and Clinical Sciences L Sacco University of Milan Milano Italy; 6 Unit of Geriatrics, Department of Medicine Biomedical Campus of Rome Roma Italy; 7 see Acknowledgments Segrate (MI) Italy

**Keywords:** SARS-CoV-2, COVID-19, smoking habit, dose-response relationship, nasopharyngeal swab testing, infection severity, web-based survey, self-reported, cross-sectional design

## Abstract

**Background:**

Several studies have reported a low prevalence of current smoking among hospitalized COVID-19 cases; however, no definitive conclusions can be drawn.

**Objective:**

We investigated the association of tobacco smoke exposure with nasopharyngeal swab (NPS) test results for SARS-CoV-2 infection and disease severity accounting for possible confounders.

**Methods:**

The nationwide, self-administered, cross-sectional web-based Italian National Epidemiological Survey on COVID-19 (EPICOVID19) was administered to an Italian population of 198,822 adult volunteers who filled in an online questionnaire between April 13 and June 2, 2020. For this study, we analyzed 6857 individuals with known NPS test results. The associations of smoking status and the dose-response relationship with a positive NPS test result and infection severity were calculated as odds ratios (ORs) with 95% CIs by means of logistic and multinomial regression models adjusting for sociodemographic, clinical, and behavioral characteristics.

**Results:**

Out of the 6857 individuals (mean age 47.9 years, SD 14.1; 4516/6857, 65.9% female), 63.2% (4334/6857) had never smoked, 21.3% (1463/6857) were former smokers, and 15.5% (1060/6857) were current smokers. Compared to nonsmokers, current smokers were younger, were more educated, were less affected by chronic diseases, reported COVID-19–like symptoms less frequently, were less frequently hospitalized, and less frequently tested positive for COVID-19. In multivariate analysis, current smokers had almost half the odds of a positive NPS test result (OR 0.54, 95% CI 0.45-0.65) compared to nonsmokers. We also found a dose-dependent relationship with tobacco smoke: mild smokers (adjusted OR [aOR] 0.76, 95% CI 0.55-1.05), moderate smokers (aOR 0.56, 95% CI 0.42-0.73), and heavy smokers (aOR 0.38, 95% CI 0.27-0.53). This inverse association also persisted when considering the severity of the infection. Current smokers had a statistically significantly lower probability of having asymptomatic (aOR 0.50, 95% CI 0.27-0.92), mild (aOR 0.65, 95% CI 0.53-0.81), and severe infections (aOR 0.27, 95% CI 0.17-0.42) compared to those who never smoked.

**Conclusions:**

Current smoking was negatively associated with SARS-CoV-2 infection with a dose-dependent relationship. Ad hoc experimental studies are needed to elucidate the mechanisms underlying this association.

**Trial Registration:**

ClinicalTrials.gov NCT04471701; https://clinicaltrials.gov/ct2/show/NCT04471701

## Introduction

In June 2020 the World Health Organization released a report warning that smoking habits could be associated with adverse COVID-19 prognosis [1]. Based on extensive evidence, the report highlighted the negative impact of tobacco use on lung health and its causal association with both viral and bacterial respiratory infections [1]. In humans, the binding pathway of the spike protein with angiotensin-converting enzyme 2 (ACE2) constitutes a cell-binding site for the SARS-CoV-2 spike protein [2]. ACE2 was found to be upregulated in the small airway epithelia of smokers [3], which partially explains the increased risk of severe COVID-19 in this subpopulation [4].

However, studies from several European and non-European countries, including China [5], the United States [6], Mexico [7], Israel [8], France [9], the United Kingdom [10], and Italy [11-13], have shown an unusually low proportion of active smokers among hospitalized patients with respect to the general population. Moreover, a negative association between current smoking prevalence and COVID-19 occurrence at the population level was found in an ecological study performed in 38 European countries [14] and in a few nonhospitalized populations [15-17]. Possible biological mechanisms have been proposed to explain the counterintuitive underrepresentation of smokers among COVID-19 patients [18,19], strengthening the concept of the “smoker’s paradox” [20,21].

Nevertheless, possible explanations for these findings could be due to biases in the available data. Considering the emergency of the epidemic, it has been suggested that the smoking status and smoking history of patients, including the duration, the quantity, or the time from possible smoking cessation, may not have been accurately recorded or some patients may not have been able to report their smoking habits, leading to a misclassification of smoking status. Moreover, the ascertainment of smoking exposure has not been supported by the use of objective biomarkers [19,20], or smokers may be taking medications or exhibiting behaviors that induce some protection against COVID-19 [22]. Finally, the majority of the studies conducted to date were performed in clinical settings without a detailed evaluation of possible confounders (ie, area of residence and socioeconomic factors), and in meta-analyses, heterogeneous studies were pooled together [23].

Bearing these considerations in mind, in this study we postulated that smoking habits were associated with both SARS-CoV-2 infection and disease severity in the general population, with a dose-response relationship independent of confounding factors not considered in previous studies. To verify this hypothesis, we used data from the self-administered web-based EPICOVID19 (Italian National Epidemiological Survey on COVID-19) with the following aims: (1) to evaluate the frequency distribution of sociodemographic, clinical, and behavioral characteristics among participants according to smoking status and (2) to investigate the cross-sectional association of smoking patterns (ie, intensity and duration) with SARS-CoV-2 nasopharyngeal swab (NPS) test results and infection severity, taking into account a wide number of potential confounding factors.

## Methods

### Study Design, Setting, and Population

**Figure 1:**
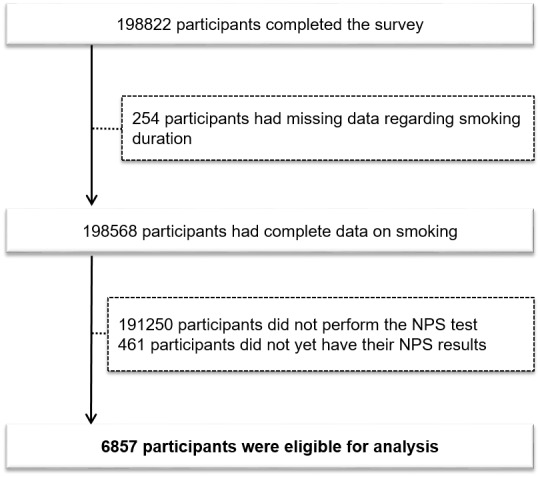
Flowchart of participant recruitment and eligibility for the EPICOVID19 (Italian National Epidemiological Survey on COVID-19) study. NPS: nasopharyngeal swab.

The study population was derived from the EPICOVID19 national internet-based survey [24] that was conducted using a cross-sectional research design in a self-selected sample of adult volunteers living in Italy during the lockdown from March to May 2020; during this same period, the total confirmed COVID-19 infected cases in Italy were 233,515 [25]. The study procedures were described elsewhere [24]. Briefly, the link to the web-based survey was implemented using the EUSurvey management tool. The survey was uploaded and shared from April 13 to June 2, 2020, via several channels: emails, social media platforms (ie, Facebook, Twitter, Instagram, and WhatsApp), press releases, internet pages, local radio and TV stations, institutional websites, mailing lists, and the study website. The inclusion criteria to take part in the survey were being aged 18 years or older; having access to a mobile phone, computer, or tablet with internet connectivity; and providing online consent to participate in the study. Out of the 198,822 participants who provided consent to participate and completed the online survey, 254 had missing data about smoking duration; 191,250 did not perform the NPS test; and 461 did not yet know their NPS test result, leading to a final sample of 6857 (3.4%) participants for this study’s analysis (Figure 1).

Compared to the people who were excluded (n=191,250) for not having performed the NPS test, those who were included (N=6857) in the analysis were more likely to be female, less educated, employed, employed in white collar jobs, health care professionals, residents in northern regions, affected by chronic diseases, and frequently vaccinated for flu and pneumococcal disease. They were also more likely to report symptoms, be frequently hospitalized, never have smoked, be living in big suburbs or cities and crowded houses, have frequently reported contacts with COVID-19 cases and called the emergency numbers, and have had a lower self-perceived health status (Table S1 in Multimedia Appendix 1).

### Ethical Approval

The Ethics Committee of the Istituto Nazionale per le Malattie Infettive IRCCS Lazzaro Spallanzani (protocol No. 70, 12/4/2020) approved the EPICOVID19 study protocol. When participants first accessed the web-based platform, they were informed about the study and its purpose, the data to be collected, and the methods of storage; they then filled in the informed consent form. Participation was voluntary and no compensation was expected for respondents. The planning, conduct, and reporting of the study were in line with the Declaration of Helsinki, as revised in 2013. Data were handled and stored following the European Union General Data Protection Regulation (EU GDPR) 2016/679, and data transfer was safeguarded by encrypting and decrypting data and password protection. The study was registered at ClinicalTrials.gov (NCT04471701).

### Data Collection and Variables Definition

The EPICOVID19 study was established as a collaborative project of a working group including epidemiologists, physicians who were experts in infectious diseases, biostatisticians, and public health professionals to improve SARS-CoV-2–related knowledge. To guarantee maximal comparability with other studies, several questions were defined based on standardized and validated questionnaires, as described elsewhere in detail [24,26,27]. The participants were asked to complete an anonymous 38-item questionnaire (Multimedia Appendix 2) that mainly contained mandatory and closed questions divided into six sections: sociodemographics, clinical features, personal characteristics, behaviors before the lockdown, lifestyles, and behaviors following the lockdown (Multimedia Appendix 1).

### Smoke Exposure

Several questions on present and past smoking habits were asked in the questionnaire. These included smoking status defined as *never smoked* (ie, persons who had never smoked regularly or had smoked less than 100 cigarettes), *former smokers* (ie, regular smokers who have smoked at least 100 cigarettes during their lifetime and did not smoke at the time of the survey), and *current smokers* [28]. To explore the dose-response effect, we created a variable by collapsing data on smoking status and smoking duration in years as follows: *former smokers* (ie, categorized for the smoking duration of ≤10 years or >10 years), *current smokers grouped in with mild smokers* (ie, ≤10 cigarettes/day for <15 years), *moderate smokers* (ie, ≤10 cigarettes/day for ≥15 years or >10 cigarettes/day for <15 years), and *heavy smoker*s (ie, >10 cigarettes/day for ≥15 years).

### Main Outcomes

We investigated two different outcomes: (1) positive result for the NPS molecular test and (2) SARS-CoV-2 infection severity by combining information from the NPS test, symptoms, and hospitalization for COVID-19 defined as follows:

- No infection — negative NPS test
- Asymptomatic infection — positive NPS test without COVID-19–like symptoms excluding pneumonia
- Mild infection — positive NPS test with at least one COVID-19–like symptom excluding pneumonia
- Severe infection — positive NPS test with pneumonia and/or hospitalization for COVID-19.

### Statistical Analysis

The continuous variables were represented as means and SDs and the categorical variables as counts and percentages. Continuous and categorical data according to smoking status were compared using one-way analyses of variance and chi-square tests, respectively. To explore the association between smoking habits, positive versus negative NPS test results, and the 4-level infection-severity dependent variable (ie, no infection, asymptomatic infection, mild infection, and severe infection), logistic regression and multinomial regression models were used to estimate the odds ratios (ORs) and 95% CIs. The first model was adjusted for age and sex. In the fully adjusted model, we further controlled for variables that were considered potential confounders, such as education, occupation, area of residence, heart diseases, lung diseases, hypertension, metabolic diseases, contact with suspected or confirmed COVID-19 cases, living area, crowding index, and living with at-risk cohabitants. Models were applied considering the smoking status and the dose-response relationship as exposures separately. We explored our data for potential effect modification by sex, age, and education by adding cross-product terms of these variables to the regression models. When heterogeneity was present, stratum-specific estimates were evaluated. Three sensitivity analyses were performed to evaluate whether the effect of smoking on SARS-CoV-2 infection was primarily due to the current amount of cigarettes smoked and/or to the smoking history during the lifespan. In the first sensitivity analysis, we categorized current smokers based on years of smoking into the following groups: <15 years, 15-30 years, and >30 years. The second sensitivity analysis explored the association between the number of cigarettes smoked, categorized as ≤10 cigarettes/day or >10 cigarettes/day, and the NPS test result. The third sensitivity analysis repeated the analysis by calculating the pack-years of smoking. We assigned a median number of cigarettes per day to each current smoking category (5 for <10 cigarettes/day; 15 for 10-20 cigarettes/day; 25 for >20 cigarettes/day), then we multiplied the number of packs per day (1 pack = 20 cigarettes) by the number of years the person had smoked; finally, we categorized the variable into tertiles. All statistical analyses were performed using Stata 15.0 (StataCorp LLC), and a two-sided *P* value <.05 was considered statistically significant.

## Results

### Characteristics of the Participants

The participants’ characteristics regarding their smoking status are summarized below. The mean age of the whole sample was 47.9 (SD 14.1) years, 65.9% (4516/6857) were females, and 70.5% (4834/6857) had a university degree or higher. Out of 6857 individuals, 63.2% (n=4334) had never smoked, 21.3% (n=1463) were former smokers, and 15.5% (n=1060) were current smokers. A total of 24.7% (1691/6857) of the participants had a positive NPS test; among them, 9.2% (156/1691) were asymptomatic, 62.0% (1049/1691) had a mild infection, and 28.7% (486/1691) reported conditions compatible with a severe infection. Compared with those who never smoked, current smokers were younger, had higher educational levels, more frequently worked as employers, were health care professionals, and were frequently residents in central and southern regions (Table 1).

**Table 1:** Sociodemographic characteristics of study participants with known molecular test results by smoking status in Italy, from April 13 to June 2, 2020 (N=6857).

Sociodemographic characteristics		Never smoked (n=4334)	Former smoker (n=1463)	Current smoker (n=1060)	*P* value (current vs never)		*P* value (overall)		All participants (N=6857)	
All participants, n (%)		4334 (63.2)	1463 (21.3)	1060 (15.5)	N/A^a^		N/A		6857 (100)	
Sex (female), n (%)		2991 (69.0)	807 (55.2)	420 (70.1)	.42		<.001		4516 (65.9)	
Age (years), mean (SD)		47.7 (14.7)	50.5 (13.1)	45.0 (12.4)	<.001		<.001		47.9 (14.1)	
European ethnicity, n (%)		4281 (98.8)	1458 (99.7)	1052 (99.3)	.20		.01		6791 (99.0)	
**Education, n (%)**						.01		.01		
	Illiterate or primary school	359 (8.3)	96 (6.6)	30 (5.0)					525 (7.7)	
	Middle or high school	893 (20.6)	347 (23.7)	126 (21.0)					1498 (21.9)	
	University or postgraduate degree	3082 (71.1)	1020 (69.7)	443 (74.0)					4834 (70.5)	
**Employment status, n (%)**						<.001		<.001		
	Employed	3626 (83.7)	1223 (83.6)	548 (91.5)					5811 (84.8)	
	Student	125 (2.9)	18 (1.2)	19 (3.2)					172 (2.5)	
	Unemployed	63 (1.5)	28 (1.9)	8 (1.3)					106 (1.6)	
	Retired	291 (6.7)	144 (9.8)	8 (1.3)					459 (6.7)	
	Other	229 (5.3)	50 (3.4)	16 (2.7)					309 (4.5)	
**Occupational cluster^b^, n (%)**						.07		<.001		
	White collar	3428 (79.1)	1173 (80.2)	804 (75.9)					5405 (78.8)	
	Blue collar	58 (1.3)	37 (2.5)	17 (1.6)					112 (1.6)	
	Other	848 (19.6)	253 (17.3)	239 (22.6)					1340 (19.5)	
Health professional		2164 (49.9)	680 (46.5)	628 (59.3)	<.001		<.001		3472 (50.6)	
**Italian area of residence, n (%)**						.01		.01		
	Northern	3318 (76.6)	1084 (74.1)	755 (71.2)					5157 (75.2)	
	Central	686 (15.8)	231 (15.8)	204 (19.3)					1121 (16.4)	
	Southern	320 (7.8)	144 (9.8)	98 (9.3)					562 (8.2)	
	Other	10 (0.2)	4 (0.3)	3 (0.3)					17 (0.3)	

^a^N/A: not applicable; *P* value was not calculated.

^b^White collar occupations include legislators, senior officials and managers, professionals, technicians, associate professionals, clerks and service workers, and shop and market sales workers; blue collar occupations include skilled agricultural and fishery workers, craft and related trades workers, plant and machine operators and assemblers, elementary occupations, others including armed forces, and unspecified occupations.

Current smokers were less affected by heart diseases (ie, cardiovascular disease [CVD]), hypertension, oncological diseases, and allergies compared to those who never smoked. They were less dependent in their daily activities, were less frequently vaccinated for flu and pneumococcal infections, less frequently took thyroid drugs and supplements, and more frequently took anti-inflammatory drugs. Smokers reported COVID-19–like symptoms less frequently, such as fever, olfactory and taste disorders, shortness of breath, cough, and pneumonia; they were less frequently hospitalized for COVID-19, had fewer NPS positive tests, and were less likely to be infected by SARS-CoV-2 in comparison with those who never smoked (Table 2).

**Table 2:** Clinical features of study participants with known molecular test results by smoking status in Italy, from April 13 to June 2, 2020 (N=6857).

Clinical features			Never smoked (n=4334)		Former smoker (n=1463)		Current smoker (n=1060)			*P* value (current vs never)		*P* value (overall)			All participants (N=6857)	
All participants, n (%)			4334 (63.2)		1463 (21.3)		1060 (15.5)			N/A^a^		N/A			6857 (100)	
**Self-reported diseases, n (%)**																
	Lung diseases	340 (7.8)		130 (8.9)		77 (7.3)		.53			.29			547 (8.0)		
	Heart diseases	196 (4.5)		76 (5.2)		26 (2.5)		.01			.01			298 (4.4)		
	Hypertension and/or medications	723 (16.7)		326 (22.3)		143 (13.5)		.01			<.001			1192 (17.4)		
	Oncological diseases	138 (3.2)		67 (4.6)		16 (1.5)		.01			.001			221 (3.2)		
	Liver diseases	39 (0.9)		14 (1.0)		6 (0.6)		.28			.52			59 (0.9)		
	Renal diseases	52 (1.2)		14 (1.0)		10 (0.9)		.48			.64			76 (1.1)		
	Metabolic diseases and/or medications	238 (5.5)		86 (5.9)		47 (4.4)		.17			.27			371 (5.4)		
	Depression or anxiety and/or medications	505 (11.7)		167 (11.4)		122 (11.5)		.90			.97			794 (11.6)		
	Immune system diseases	431 (9.9)		146 (10.0)		88 (8.3)		.10			.25			665 (9.7)		
	Surgical procedures last year	168 (3.9)		82 (5.6)		38 (3.6)		.66			.01			288 (4.2)		
	Transplants	12 (0.3)		6 (0.4)		0 (0)		.09			.13			18 (0.3)		
	Allergies	786 (18.1)		223 (15.2)		163 (15.4)		.04			.01			1172 (17.1)		
	Dependency in daily activities	209 (4.8)		15 (1.0)		9 (0.9)		<.001			<.001			233 (3.4)		
	Flu shot during last autumn	1542 (35.6)		489 (33.4)		273 (25.8)		<.001			<.001			2304 (33.6)		
	Antipneumococcal vaccine in the last 12 months	219 (5.1)		73 (5.0)		37 (3.5)		.03			.10			329 (4.8)		
**Self-reported medications, n (%)**																
	Aspirin	192 (4.4)		131 (9.0)		46 (4.3)		.10			<.001			369 (5.4)		
	Cholesterol treatment drugs	252 (5.8)		161 (11.0)		69 (6.5)		.39			<.001			482 (7.0)		
	Oncological drugs	42 (1.0)		23 (1.6)		6 (0.6)		.21			.04			71 (1.0)		
	Corticosteroids	95 (2.2)		39 (2.7)		24 (2.3)		.89			.58			158 (2.3)		
	Thyroid drugs	369 (8.5)		131 (9.0)		64 (6.0)		.01			.02			564 (8.2)		
	Anti-inflammatory drugs	222 (5.1)		108 (7.4)		101 (9.5)		<.001			<.001			431 (6.3)		
	Supplements or vitamins	928 (21.4)		304 (20.8)		190 (17.9)		.01			.04			1422 (20.7)		
**Self-reported symptoms, n (%)**																
	Fever	1221 (28.2)		491 (33.6)		184 (17.4)		<.001			<.001			1896 (27.7)		
	Headache	1594 (36.8)		570 (39.0)		397 (37.5)		.68			.33			2561 (37.4)		
	Muscle or bone pain	1476 (34.1)		563 (38.5)		340 (32.1)		.22			<.001			2379 (34.7)		
	Olfactory and taste disorders	903 (20.8)		365 (25.0)		180 (17.0)		.01			<.001			1448 (21.1)		
	Shortness of breath	643 (14.8)		264 (18.1)		127 (12.0)		.02			<.001			1034 (15.1)		
	Chest pain	596 (13.8)		224 (15.3)		144 (13.6)		.89			.30			964 (14.1)		
	Heart palpitations	572 (13.2)		185 (12.7)		118 (11.1)		.07			.19			875 (12.8)		
	Gastrointestinal disturbances	1210 (27.9)		441 (30.1)		275 (25.9)		.20			.06			1926 (28.1)		
	Conjunctivitis	527 (12.2)		174 (11.9)		117 (11.0)		.31			.60			818 (11.9)		
	Sore throat or rhinorrhea	1579 (36.4)		558 (38.1)		392 (37.0)		.74			.50			2529 (36.9)		
	Cough	1537 (35.5)		536 (36.6)		294 (27.7)		<.001			<.001			2367 (34.5)		
	Pneumonia	354 (8.2)		170 (11.6)		32 (3.0)		<.001			<.001			556 (8.1)		
	No symptoms	1154 (26.6)		321 (21.9)		308 (29.1)		.11			<.001			1783 (26.0)		
	Hospitalized for COVID-19	319 (7.4)		175 (12.0)		33 (3.1)		<.001			<.001			527 (7.7)		
	Positive NPS^b^ test result	1124 (25.9)		407 (27.8)		160 (15.1)		<.001			<.001			1691 (24.7)		
**Infection severity^c^, n (%)**									N/A				<.001			
	No infection	3210 (74.1)		1056 (72.2)		900 (84.9)								5166 (75.3)		
	Asymptomatic	117 (2.7)		27 (1.9)		12 (1.1)								156 (2.3)		
	Mild	697 (16.1)		225 (15.4)		127 (12.0)								1049 (15.3)		
	Severe	310 (7.2)		155 (10.6)		21 (2.0)								486 (7.1)		

^a^N/A: not applicable; *P* value was not calculated.

^b^NPS: nasopharyngeal swab.

^c^No infection: negative NPS test result; asymptomatic infection: positive NPS test result without COVID-19–like symptoms excluding pneumonia; mild infection: positive NPS test result with at least one COVID-19–like symptom excluding pneumonia; and severe infection: positive NPS test with pneumonia and/or hospitalization for COVID-19

Current smokers lived less frequently with cohabitants who were at risk of COVID-19 infection; after the lockdown, they more frequently went out and used public transport, they contacted the emergency number less frequently, and they were more afraid of themselves or family members becoming infected than were nonsmokers (Table 3).

In comparison with people who never smoked and current smokers, former smokers were significantly older; retired; more affected by chronic conditions, such as heart diseases and hypertension; and more frequently took aspirin, drugs for lowering cholesterol, and oncological and thyroid drugs. They reported COVID-19–like symptoms less frequently and were more likely to be hospitalized for COVID-19.

**Table 3:** Behavioral characteristics of study participants with known molecular test results by smoking status in Italy, from April 13 to June 2, 2020 (N=6857).

Behavioral characteristics			Never smoked (n=4334)	Former smoker (n=1463)	Current smoker (n=1060)	*P* value (current vs never)		*P* value (overall)		All participants (N=6857)	
All participants, n (%)			4334 (63.2)	1463 (21.3)	1060 (15.5)	N/A^a^		N/A		6857 (100)	
**Housing conditions, n (%)**											
	**Traffic near house**						.25		.17		
		Low	1880 (43.4)	655 (44.8)	461 (43.5)					2996 (43.7)	
		Moderate	1499 (34.6)	525 (35.9)	388 (36.6)					2412 (35.2)	
		High	955 (22.0)	283 (19.3)	211 (19.9)					1449 (21.1)	
	Cohabitants at risk^b^		934 (21.6)	250 (17.1)	182 (17.2)	.01		<.001		1366 (19.9)	
	**Residence area**						.32		.25		
		Countryside	492 (11.4)	164 (11.2)	136 (12.8)					792 (11.6)	
		Small town	1797 (41.5)	609 (41.6)	411 (38.8)					2817 (41.1)	
		Suburbs: >100,000 inhabitants	772 (17.8)	233 (15.9)	198 (18.7)					1203 (17.5)	
		City or town: >100,000 inhabitants	1273 (29.4)	457 (31.2)	315 (29.7)					2045 (29.8)	
	**Household crowding index^c^**						.33		.10		
		Low	3941 (90.9)	1354 (92.6)	974 (91.9)					6269 (91.4)	
		Middle	387 (8.9)	105 (7.2)	86 (8.1)					578 (8.4)	
		High	6 (0.1)	4 (0.3)	0 (0)					10 (0.2)	
**Behaviors before the lockdown, n (%)**											
	**Number of daily contacts**						.17		.01		
		<10	738 (17.0)	293 (20.0)	162 (15.3)					1193 (17.4)	
		≥10	3596 (83.0)	1170 (80.0)	898 (84.7)					5664 (82.6)	
	**Physical activity**						.88		<.001		
		>2.5 h/week	1099 (25.4)	449 (30.7)	275 (25.9)					1826 (26.6)	
		10 min/week to 2.5 h/week	1870 (43.1)	631 (43.1)	449 (42.4)					2950 (43.0)	
		<10 min/week	1363 (31.5)	383 (26.2)	336 (31.7)					2082 (30.4)	
**Behaviors after the lockdown, n (%)**											
	Contact with COVID-19 cases^d^		3118 (71.9)	989 (67.6)	754 (71.1)	.60		.01		4861 (70.9)	
	**Weekly outings**						<.001		<.001		
		Never	1043 (24.1)	364 (24.9)	140 (13.2)					1547 (22.6)	
		1-3	1083 (25.0)	417 (28.5)	290 (27.4)					1790 (26.1)	
		≥4	2208 (51.0)	682 (46.6)	630 (59.4)					3520 (51.3)	
	**Use of public transport**						.05		.05		
		Never	4186 (96.6)	1423 (97.3)	1008 (95.1)					6617 (96.5)	
		1-3 times/week	62 (1.4)	18 (1.2)	25 (2.4)					105 (1.5)	
		≥4 times/week	86 (2.0)	22 (1.5)	27 (2.6)					135 (2.0)	
**Personal characteristics, n (%)**											
	**Contacted emergency number**						<.001		<.001		
		No	2323 (53.6)	722 (49.4)	676 (63.8)					3721 (54.3)	
		No, but I went to a hospital on my own initiative	103 (2.4)	47 (3.2)	18 (1.7)					168 (2.5)	
		Yes, and they did not suggest that I self-isolate	235 (5.4)	88 (6.0)	72 (6.8)					395 (5.8)	
		Yes, and they suggested that I self-isolate	1361 (31.4)	448 (30.6)	239 (22.6)					2048 (29.9)	
		Yes, I was sent to a hospital	312 (7.2)	158 (10.8)	55 (5.2)					525 (7.7)	
	**Self-perceived health status**						.81		.59		
		Good	3493 (80.6)	1155 (79.0)	863 (81.4)					5511 (80.4)	
		Adequate	769 (17.7)	282 (19.3)	179 (16.9)					1230 (17.9)	
		Bad	72 (1.7)	26 (1.8)	18 (1.7)					116 (1.7)	
	**Afraid to be infected**						.02		.01		
		No	1556 (35.9)	521 (35.6)	401 (37.8)					2478 (36.1)	
		Neutral	918 (21.2)	253 (17.3)	184 (17.4)					1355 (19.8)	
		Yes	1860 (42.9)	689 (47.1)	475 (44.8)					3024 (44.1)	
	**Afraid for family members**						<.001		<.001		
		No	669 (15.4)	251 (17.2)	179 (16.9)					1099 (16.0)	
		Neutral	514 (11.9)	118 (8.1)	64 (6.0)					696 (10.2)	
		Yes	3151 (72.7)	1094 (74.8)	817 (77.1)					5062 (73.8)	

^a^N/A: not applicable; *P* value was not calculated.

^b^This includes elderly persons or anyone who is immunocompromised or has chronic disease conditions.

^c^Number of cohabitants per number of rooms.

^d^Suspected or confirmed COVID-19 cases.

### Association Analyses

**Figure 2:**
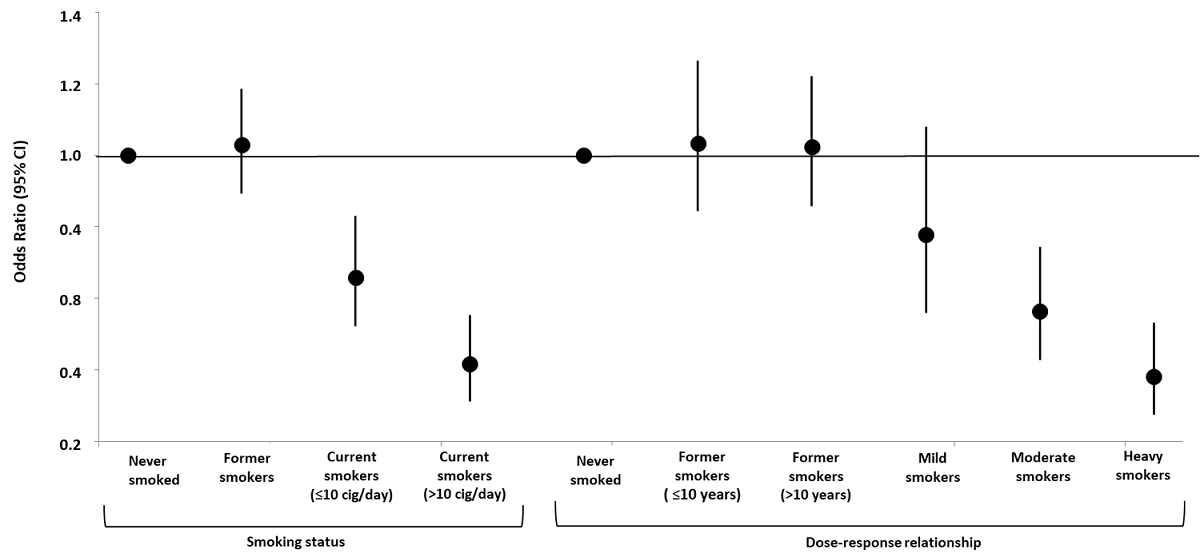
Adjusted odds ratios and relative 95% CIs for smoking status, intensity, and duration (N=6857). Odds ratios were adjusted for age, sex, education, occupation, area of residence, heart diseases, lung diseases, hypertension, metabolic and oncological diseases, contact with confirmed or suspected COVID-19 cases, living area, crowding index, and living with at-risk cohabitants. Dots and vertical lines indicate adjusted odds ratios and 95% CIs, respectively. Mild smokers: ≤10 cigarettes/day for <15 years; moderate smokers: ≤10 cigarettes/day for ≥15 years or >10 cigarettes/day for <15 years; heavy smokers: >10 cigarettes/day for ≥15 years. cig: cigarettes.

Table S2 in Multimedia Appendix 1 and Figure 2 show the logistic regression results for positive NPS tests. In the age- and sex-adjusted model, current smoking was significantly inversely associated with a positive NPS test (OR 0.54, 95% CI 0.45-0.65), with *never smoked* as the reference category. Results did not change when potential confounders were accounted for in the fully adjusted model (adjusted OR [aOR] 0.54, 95% CI 0.44-0.65). Being a former smoker was not associated with a positive NPS test (aOR 1.03, 95% CI 0.90-1.19), even when we considered the dose-response relationship and the lifetime smoking duration (≤10 years and >10 years). The aOR for testing positive was 0.76 in mild smokers (95% CI 0.55-1.05), although not statistically significant; 0.56 in moderate smokers (95% CI 0.42-0.73); and 0.38 in heavy smokers (95% CI 0.27-0.53), suggesting a dose-response relationship between smoking habit and NPS test result.

Table 4 reports the association between smoking status and infection severity. Current smokers had a statistically significant lower probability of having an asymptomatic infection (aOR 0.50, 95% CI 0.27-0.92), mild infection (aOR 0.65, 95% CI 0.53-0.81), and severe infection (aOR 0.27, 95% CI 0.17-0.42) compared to people who never smoked. The inverse dose-dependent relationship also persisted when considering the gravity of the infection, showing a gradient of association across smoking patterns. Since we found a significant interaction between smoking status and age (*P*=.001), we created a 6-level variable by combining age—dichotomized into ≤48 years and >48 years (median) groups—and smoking status. Compared to people who never smoked and were 48 years of age or younger, people who never smoked or former smokers who were over 48 years of age had a 1.5-fold and 1.7-fold higher probability of a positive NPS test, respectively.

**Figure 3:**
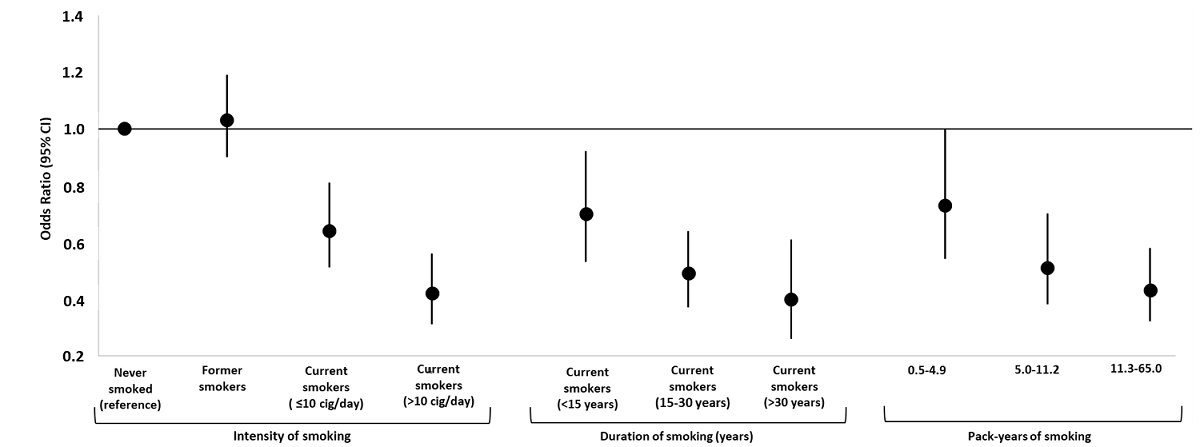
Adjusted odds ratios for positive SARS-CoV-2 tests by smoke-related variables: intensity, duration, and pack-years of smoking (N=6857). Odds ratios were adjusted for age, sex, education, occupation, area of residence, heart diseases, lung diseases, hypertension, metabolic and oncological diseases, contact with COVID-19 cases, living area, crowding index, and living with at-risk cohabitants. Dots and vertical lines indicate adjusted odds ratios and 95% CIs, respectively. cig: cigarettes.

The odds were reduced by 33% and 42% in current smokers aged 48 years or younger and those more than 48 years of age, respectively (Table S3 in Multimedia Appendix 1). In sensitivity analyses, considering *never smoked* as the reference category, we found that the inverse relationship between smoking and a positive NPS test was stronger in heavy smokers (>10 cigarettes/day; aOR 0.42, 95% CI 0.31-0.56), in long-term smokers (smoked for >30 years; aOR 0.40, 95% CI 0.26-0.61), and in those in the highest pack-years category (pack-years 11.3-65; aOR 0.43, 95% CI 0.32-0.58). In moderate smokers (≤10 cigarettes/day; aOR 0.64, 95%CI 0.51-0.81), more recent current smokers (smoked for <15 years; aOR 0.70, 95% CI 0.53-0.92), and those in the lowest category of pack-years of smoking (pack-years 0.5-4.9; aOR 0.73, 95% CI 0.54-1.00), the odds reduction was lower (Figure 3 and Table S4 in Multimedia Appendix 1).

**Table 4:** Odds ratios (ORs)^a^ of SARS-CoV-2 severity^b^ by smoking habit (N=6857).

Smoking habit			No infection (n=5166), n (%)		Asymptomatic infection (n=156)				Mild infection (n=1049)					Severe infection (n=486)		
					OR (95% CI)		n (%)		OR (95% CI)		n (%)		OR (95% CI)			n (%)
Total participants (N=6857)			5166 (75.3)		N/A^c^		156 (2.3)		N/A		1049 (15.3)		N/A			486 (7.1)
**Smoking status**																
	Never smoked	3210 (62.1)		1 (reference)		117 (75.0)		1 (reference)		697 (66.4)		1 (reference)			310 (63.8)	
	Former smokers	1056 (20.4)		0.78 (0.50-1.21)		27 (17.3)		0.99 (0.84-1.18)		225 (21.5)		1.20 (0.97-1.50)			155 (31.9)	
	Current smokers	900 (17.4)		0.50 (0.27-0.92)		12 (7.7)		0.65 (0.53-0.81)		127 (12.1)		0.27 (0.17-0.42)			21 (4.3)	
**Dose-response relationship**																
	Never smoked	3210 (62.1)		1 (reference)		117 (75.0)		1 (reference)		697 (66.4)		1 (reference)			310 (63.8)	
	Former smokers (≤10 years)	487 (9.4)		0.84 (0.42-1.71)		9 (5.8)		1.00 (0.79-1.27)		99 (9.4)		1.22 (0.88-1.69)			50 (10.3)	
	Former smokers (>10 years)	569 (11.0)		0.74 (0.44-1.27)		18 (11.5)		0.98 (0.79-1.22)		126 (12.0)		1.20 (0.92-1.55)			105 (21.6)	
	Mild smokers^d^	249 (4.8)		1.16 (0.41-3.29)		4 (2.6)		0.84 (0.59-1.18)		42 (4.0)		0.23 (0.07-0.73)			3 (0.6)	
	Moderate smokers^e^	365 (7.1)		0.42 (0.15-1.15)		4 (2.6)		0.67 (0.49-0.91)		52 (5.0)		0.35 (0.19-0.66)			11 (2.3)	
	Heavy smokers^f^	286 (5.5)		0.36 (0.13-0.99)		4 (2.6)		0.50 (0.34-0.72)		33 (3.2)		0.20 (0.09-0.43)			7 (1.4)	

^a^ORs were adjusted for age, sex, education, occupation, area of residence, heart diseases, lung diseases, hypertension, metabolic and oncological diseases, contact with COVID-19 cases, living area, crowding index, and living with at-risk cohabitants.

^b^No infection: negative nasopharyngeal swab (NPS) test (reference category); asymptomatic infection: positive NPS test without symptoms; mild infection: positive NPS test with at least one symptom; and severe infection: positive NPS test with pneumonia and/or hospitalization for COVID-19.

^c^N/A: not applicable.

^d^Mild smokers: ≤10 cigarettes/day for <15 years.

^e^Moderate smokers: ≤10 cigarettes/day for ≥15 years or >10 cigarettes/day for <15 years.

^f^Heavy smokers: >10 cigarettes/day for ≥15 years.

## Discussion

### Principal Findings

This study evaluated the association between smoking habits and the odds of receiving positive SARS-CoV-2 molecular tests and infection severity in an Italian adult population recruited online during the first national lockdown. We found that current smoking was associated with a significant risk reduction of having a positive SARS-CoV-2 test and of developing a severe infection in a dose-response relationship, even after taking into account all the available confounding factors.

In our sample, the percentage of positive tests in participants was 24.7% (1691/6857), close to the positive test ratio shown by Romagnani and colleagues, who reported, at the beginning of April 2020, an overall percentage of positive tests of 18.6% for Italy, with a marked regional difference ranging from 38.5% in Lombardy to 7.5% in Lazio [29]. The relatively high percentage of positive tests reflects the initial phase of the pandemic spread, during which, in Italy, molecular tests were reserved for clinically relevant cases. This is in keeping with the low percentage of asymptomatic subjects in our sample: 2.3% of the overall evaluated sample and 9.2% among confirmed SARS-CoV-2 infection cases. Although 70.5% of participants had a university degree or higher and the female gender was predominant (65.9%), the prevalence of smoking habits in our sample was quite similar to that of the Italian general population. Indeed, we found that 63.2% of the included participants had never smoked, 21.3% were former smokers, and 15.5% were current smokers. In Italy, the prevalence of former smokers is 23.0%, while active smokers represent 18.4% of the population and those who never smoked represent 57.4% of the population [30].

When compared to those who never smoked, current smokers had a lower prevalence of chronic conditions (50.8% vs 55.4%), including those known to be influenced by smoking habits, such as CVD (2.5% vs 4.5%) and hypertension (13.5% vs 16.7%). Former smokers were older and more frequently retired compared to those who never smoked and they were, as expected, more affected by chronic diseases, such as CVD (5.2%) and hypertension (22.3%). This finding is consistent with the successful smoking cessation achieved by subjects affected by hypertension and myocardial infarction [31]. Current smokers had significantly fewer COVID-19–like symptoms and were less frequently hospitalized for COVID-19 than those who never smoked and former smokers; this is in agreement with a previous meta-analysis study showing a lower prevalence of current smokers among hospitalized COVID-19 patients [19].

We found that current smoking was associated with reduced odds of a positive NPS test by 46%. Analogously, Israel and colleagues [16] found reduced odds by 53% for the association between current smoking and fatal or severe disease in a population-based study among over 3,000,000 adults in Israel. Similar results were observed in a study on middle-aged veterans in the United States in which smokers were less likely to test positive for COVID-19 (OR 0.43), although there was no significant difference in hospitalization [17]. In a large cohort study of 17,278,392 adults from the general population in the United Kingdom, current smoking was associated with an increased risk of COVID-19–related death when controlling for age and sex. However, after adjustment for multiple adjusted covariates (eg, chronic respiratory diseases), the authors found that smoking was associated with a risk lowered by 11% [32]. A negative association between smoking prevalence and COVID-19 occurrence at the population level was also found in an ecological study conducted in 38 European countries, although the authors cautioned that this association might not imply a causal relationship [14].

In our study, we also observed a significant dose-response relationship between smoking habits and NPS test results. In the fully adjusted logistic model, mild smokers had a 24% lower probability of a positive NPS test, whereas moderate smokers and heavy smokers had, respectively, 44% and 62% lower probabilities compared to those who never smoked. Conversely, among former smokers, we did not find a significant effect of the time interval (≤10 years or >10 years) on NPS test results. A French study evaluating smoking habits among symptomatic COVID-19 inpatients and outpatients showed that, in both groups, active smokers were less frequently infected by COVID-19 when compared with the general population [33].

When we analyzed the association between smoking habits and SARS-CoV-2 infection severity, we found that active smokers were less likely to develop a severe infection. Furthermore, by evaluating participants with positive NPS test results in relation to their reported infection severity (ie, asymptomatic, mild, or severe infection), being a current smoker reduced the odds of a severe infection by 50%, 35%, and 73%, respectively. Likewise, regarding the dose-response effect found for positive NPS test results, heavy smokers showed a lower risk of developing different severity levels of SARS-CoV-2 infection, in particular severe COVID-19 (80% odds reduction). The link between smoking and infection severity is highly controversial in the literature. For example, in the previously cited meta-analysis, Farsalinos and colleagues showed that, although the risk for current smokers to be hospitalized was lower than for nonsmokers, current smokers were more likely to have an adverse outcome during their hospital admission [19]. In a population of over 2.4 million UK users of the Zoe COVID-19 Symptom Study app, Hopkinson found a statistically significant OR of 1.14 for the self-reporting of a triad of three symptoms (ie, fever, persistent cough, and shortness of breath) for current smokers; although to some extent this was also attributable to constipation or normal flu, the authors identified it as suggestive of COVID-19. On the contrary, when analyzing the stronger endpoint of a positive SARS-CoV-2 test, they observed a lower smoking rate (7.4% among positive tests vs 9.3% among negative tests), leading to a reduced aOR of 0.7 that they considered not generalizable to their general population, due to the physiological difference between tested and untested individuals [34]. In their systematic review, Vardavas and Nikitara concluded that smoking was associated with disease progression and increased adverse outcomes in COVID-19–positive patients [35]. This was the case even though, in both meta-analyses, the authors acknowledged that their studies were conducted with limited availability of data, the included studies came mostly from hospital contexts, and their analyses were not adjusted for confounding factors. Similar methodological limitations have been reported in the meta-analysis conducted by Patanavanich, who found that smoking was a risk factor for the progression of COVID-19 [36]; conversely, Lippi and Henry did not observe any association [37].

Our findings, which highlight the existence of a negative association of current smoking with SARS-CoV-2 infection and its severity, drive the focus to possible suggestive explanations. Since ACE2 is necessary for infection of cells by SARS-CoV-2 [38], the risk of contracting a severe SARS-CoV-2 infection, as well as the risk of a disadvantageous clinical outcome, could be influenced by the number of available ACE2 receptors and by the receptor-ligand interaction of ACE2 and the SARS-CoV-2 spike protein [39]. Regarding the number of ACE2 receptors, nicotine seems to have a controversial role. Recent evidence indicates that a higher number of receptors are expressed in the lung tissues of smokers [40]. On the other hand, it has been suggested that nicotine downregulates the expression and/or the activity of ACE2 [41]. However, a better disease outcome was associated with an overexpression of ACE2, which was able to compensate for the negative effects of the ACE2 downregulation induced by the cell entry of SARS-CoV-2 [42]. Moreover, a direct role of nicotine in disrupting spike protein glycosylation could, in turn, directly affect the ability of SARS-CoV-2 to infect [43]. A recent study performed on a mouse model proposes the modulation of the renin-angiotensin pathway as a therapeutic target to protect individuals with SARS-CoV-2 infection from developing acute severe lung failure and acute respiratory distress syndrome [44]. In addition to that, nicotine might exert an anti-inflammatory effect by protecting against the *cytokine-storm syndrome* that is responsible for severe SARS-CoV-2 infections [21,45]. It has also been hypothesized that the cytokine storm, with excessive production of proinflammatory molecules, could be more easily triggered in individuals who never smoked rather than in smokers, whose immune systems are more tolerant and less reactive [46].

Another potential mechanism of action involves nitric oxide produced during smoking that, due to its reported antiviral effect, might inhibit virus replication and entry in the cells [21,47].

Alternatively, from a behavioral perspective, we cannot exclude that smokers, considering themselves at higher risk of developing the disease, were more careful than those who never smoked in adopting preventative measures, such as physical distancing, hand hygiene, covering coughs, wearing masks when appropriate, having fewer social relationships, etc [48].

### Limitations and Strengths

This study has some limitations. Firstly, because of the observational nature of the study and the cross-sectional design, we cannot infer any causal relationship between smoking habits and COVID-19. In addition, misclassification of the outcome of severity may exist, since patients’ conditions in some cases—although numerically limited—might have worsened a few days after the survey, with a subsequent potential distortion of measures of association. Secondly, smoking habits were self-reported; therefore, recall bias might have led to misclassification of the exposure. Thirdly, the sample was self-selected and not entirely representative of the Italian population because it was restricted to relatively younger, female, highly educated, and relatively healthy participants; therefore, results should be treated with caution when generalized to different populations [49]. Moreover, the low percentage of asymptomatic subjects in our sample may have influenced the evaluation of the effects of smoking habits on asymptomatic subjects with positive NPS test results. Nevertheless, in a previous study, smokers were proportionally represented among asymptomatic patients [50]. Lastly, although we controlled for several potential confounders, we cannot completely rule out the possibility of residual confounding due to unmeasured factors (eg, passive smoking). Our study also has several strengths. The first one is that evaluating the effect of smoking was the primary goal of the work. The presence in our study sample of subjects from a general population with negative NPS test results allows for an internal control group (ie, individuals with negative NPS test results). The web survey reached a large sample of adults with an acceptable geographical coverage reflecting the distribution of SARS-CoV-2 infection during the study period [24] and a proportion of smokers that almost overlapped with the prevalence of current smoking in the Italian population. Finally, and contrary to previously published work, we recorded factors that are not easy to obtain from medical records of inpatients, such as exhaustive details regarding smoking habits (ie, distinguishing between former smokers, active smokers, or those who never smoked) and factors suspected to be confounders in the observed association (ie, socioeconomic status as well as clinical, behavioral, and environmental characteristics).

### Conclusions

In summary, we are aware that our findings must be carefully evaluated. This article takes as its premise the need to strengthen preventive actions against the most powerful human carcinogen known, which is also a heavy risk factor for many noncommunicable diseases [51] and disease progression in COVID-19 patients. However, we are now facing a second pandemic wave requiring the consideration of each issue that is still unresolved regarding the possible role played by smoking in COVID-19 disease. Further research on the mechanisms of interaction between tobacco smoke exposure and SARS-CoV-2 infection is warranted to fill this knowledge gap.

## Appendix

Multimedia Appendix 1 Supplementary materials.

Multimedia Appendix 2 Study survey.

## Abbreviations

ACE2: angiotensin-converting enzyme 2
aOR: adjusted odds ratio
CVD: cardiovascular disease
EPICOVID19: Italian National Epidemiological Survey on COVID-19
EU GDPR: European Union General Data Protection Regulation
NPS: nasopharyngeal swab
OR: odds ratio
