# Supplementary material for: Association Between Smoking and SARS-CoV-2 Infection: Cross-sectional Study of the EPICOVID19 Internet-Based Survey
Source: JMIR Public Health Surveill. 2021 Apr 28;7(4):e27091. doi: 10.2196/27091 (PMC8081027; doi:10.2196/27091)
Supplement: Multimedia Appendix 1 [file publichealth_v7i4e27091_app1.docx]

**SUPPLEMENTARY MATERIAL**

***Data Collection and variables definition***

1-*Socio-demographic* information included sex (male and female); age; ethnicity (European and non-European/unknown); educational level [1] (illiterate or primary school, middle or high school, and university degree or postgraduate degree); occupational status (unemployed, employed, retired, student, and other); and current/last occupation classified as white collar (including legislators, senior officials and managers, professionals, technicians and associate professionals, clerks, service workers and shop and market sales workers), blue collar (skilled agricultural and fishery workers, craft and related trades workers, plant and machine operators and assemblers and elementary occupations), and other (armed forces and other occupations) [2]. Italian Regions of residence were grouped into four areas: 1) Northern Italy (Piedmont, Lombardy, Aosta Valley, Emilia Romagna, Liguria, Trentino Alto Adige, and Friuli Venezia Giulia), 2) Central Italy (Marche, Tuscany, Lazio, and Umbria); 3) Southern Italy (Abruzzo, Molise, Campania, Sicily, Sardinia, Calabria, and Basilicata); and 4) Other (San Marino Republic, foreign countries or unknown).

2-*Clinical features* included SARS-CoV-2 related self-reported symptoms (fever of >37.5° for at least three consecutive days, headache, chest pain, myalgia, olfactory and taste disorders, shortness of breath, heart palpitations, gastrointestinal disturbances [nausea, vomiting and diarrhoea], conjunctivitis, sore throat, rhinorrhoea, and cough); pneumonia; self-reported diseases (lung diseases, heart diseases, kidney diseases, immune system diseases, tumours, liver diseases); flu vaccination during the 2019/2020 season and pneumococcal vaccination over the last 12 months; transplants, allergies, surgical procedures over the last year; past and current use of medication (aspirin, hypocholesterolaemia drugs, anti-cancer drugs, corticosteroids, thyroid drugs, anti-inflammatory drugs, supplements); being a healthcare professionals, and hospitalisation for confirmed or suspected SARS-CoV-2 infection (dichotomised as yes/no) [3,4,5]. Hypertension, metabolic diseases, depression and/or anxiety were complemented with the question on the use of anti-hypertensive, anti-diabetics, anxiety drugs and anti-depressant, respectively.

3-*Personal characteristics* included self-perceived health status [6] categorized as bad, adequate, and good; perceived risk for one’s own health and for the health of family members [7] grouped as not, neutral, and yes; and emergency number contact categorized as ‘no’, ‘yes but I went to a hospital on my own initiative’, ‘yes and they did not suggest to me self-isolation’, ‘yes and they suggested to me self-isolation’, and ‘yes I was sent to a hospital’.

4-*Behaviours before the lockdown* included housing conditions such as population density in the area of residence (city centre with >100.000 inhabitants, suburbs of cities with > 100.000 inhabitants, small town, and countryside); self-reported traffic intensity [8] (intense, moderate, low); household crowding index calculated as the total number of co-habitants divided by the total number of rooms (continuous variable was grouped into three distinct categories: <1, 1–2, and >2 residents per room) [9]; presence of at-risk co-habitants (elderly persons or anyone with immunocompromising or chronic disease conditions).

5-*Lifestyle characteristics* included daily mean number of contacts with other people (<10, 10 or more), smoking habit (details described below), and frequency of weekly physical activity (<10 minutes/week, 10-150 minutes/week, >150 minutes/week) [10].

6-*Behaviours after the lockdown* included close contacts with confirmed and/or suspected cases (dichotomised as yes/no); number of weekly outings (never, 1-3 times, and 4 times or more), and use of public transportation (never, 1-3 times/week, and 4 times/week or more).

***References***

[1] <http://uis.unesco.org/sites/default/files/documents/international-standard-classification-of-education-isced-2011-en.pdf>

[2] <https://www.eurofound.europa.eu/surveys/ewcs/2005/classification>

[3] World Health Organization & International Severe Acute Respiratory and emerging Infection Consortium. (‎2020)‎.Global COVID-19 Clinical Platform Novel Coronavirus (COVID-19)- Rapid Version, “https://www.who.int/docs/default-source/coronaviruse/who-ncov-crf.pdf?sfvrsn=84766e69_2.,” [Online].

[4] Case Record Form Instructions Severe Acute Respiratory Infection Clinical Characterization Data Tool, “ https://media.tghn.org/medialibrary/2019/06/SPRINT-SARI_CRF.V2_Dec_2017__Complete.pdf,” [Online].

[5] M. E. R. S. C. (MERS-CoV), “https://www.who.int/csr/disease/coronavirus_infections/MERS_case_investigation_questionnaire.pdf?ua=1.,” [Online].

[6] “The European Union Survey on Income and Living Conditions (EU-SILC)), and self- and own family-related risk perception have been recorded (5-point Likert response scale)”.

[7] Kim JS and .. Choi JS, “Middle East respiratory syndrome-related knowledge, preventive behaviours and risk perception among nursing students during outbreak. J Clin Nurs. 2016 Sep;25(17-18):2542-9. doi: 10.1111/jocn.13295. Epub 2016 Jun 7.”

[8] Cesaroni G, Badaloni C, Porta D, Forastiere F and .. Perucci CA, “Comparison between various indices of exposure to traffic-related air pollution and their impact on respiratory health in adults. Occup Environ Med. 2008 Oct;65(10):683-90. doi: 10.1136/oem.2007.037846. Epub 2008”

[9] Melki IS, Beydoun HA, Khogali M, Tamim H and .. Yunis KA, “National Collaborative Perinatal Neonatal Network (NCPNN). Household crowding index: a correlate of socioeconomic status and inter-pregnancy spacing in an urban setting. J Epidemiol Community Health. 2004 Jun;58(6):476-80. doi: 10.1136/jech.2003.012690.”.

[10] Centers for Disease Control and Prevention, “Strategies to Prevent Obesity and Other Chronic Diseases: The CDC Guide to Strategies to Increase Physical Activity in the Community. Atlanta: U.S. Department of Health and Human Services; 2011.

**Table S1.** **Characteristics of participants included and excluded from the analysis (N=198,107), Italy, from April 13 to June 2, 2020.**

| **Characteristics of participants** | **Excluded**  **N=191.250**  **(96.54%)** | | **Included**  **N=6.857**  **(3.46%)** | | ***P* value** | **All**  **N=198,107** | | |  |
| --- | --- | --- | --- | --- | --- | --- | --- | --- | --- |
| **Sex, female** | 113,724 | 59.5 | 4,516 | 65.9 | <.001 | | 118,240 | 59.7 | |
| Age, years, mean±SD | 47.96 | 14.7 | 47.87 | 14.1 | .64 | | 47.9 | 14.7 | |
| European ethnicity | 189457 | 99.1 | 6791 | 99.0 | .83 | | 196248 | 99.1 | |
| **Education** |  |  |  |  | <.001 | |  |  | |
| Illiterate or primary school | 11,270 | 5.9 | 525 | 7.7 |  | | 11,795 | 6.0 | |
| Middle or high school | 67084 | 35.1 | 1498 | 21.9 |  | | 68,582 | 34.6 | |
| University or post-graduate degree | 112896 | 59.0 | 26678 | 55.9 |  | | 117,730 | 59.4 | |
| **Employment status** |  |  |  |  | <.001 | |  |  | |
| Employed | 131,511 | 68.2 | 5,811 | 84.8 |  | | 136,322 | 68.8 | |
| Student | 13,217 | 6.9 | 172 | 2.5 |  | | 13389 | 6.8 | |
| Unemployed | 9049 | 4.7 | 106 | 1.6 |  | | 9155 | 4.6 | |
| Retired | 27.773 | 14.5 | 10681 | 22.4 |  | | 23232 | 14.3 | |
| Other | 10700 | 5.6 | 2753 | 5.8 |  | | 11009 | 5.6 | |
| **Occupational cluster*** |  |  |  |  | <.001 | |  |  | |
| White collar | 145,179 | 75.9 | 5,405 | 78.8 |  | | 151,584 | 76.0 | |
| Blue collar | 7,937 | 4.2 | 112 | 1.6 |  | | 8049 | 4.1 | |
| Others | 38,134 | 19.9 | 1340 | 19.5 |  | | 39,474 | 19.9 | |
| **Healthcare professionals** | 11,071 | 5.79 | 3,472 | 50.63 | <.001 | | 14,543 | 7.34 | |
| **Italian area of residence** |  |  |  |  | <.001 | |  |  | |
| Northern | 128,043 | 67.0 | 5,157 | 75.2 |  | | 133,200 | 67.2 | |
| Southern | 39,671 | 20.7 | 1,121 | 16.4 |  | | 40,792 | 20.6 | |
| Southern | 23,130 | 12.1 | 562 | 8.2 |  | | 23,692 | 12.0 | |
| Other° | 406 | 0.2 | 17 | 0.3 |  | | 423 | 0.2 | |
| **N° of diseases** |  |  |  |  | <.001 | |  |  | |
| None | 97,438 | 51.0 | 3,472 | 50.6 |  | | 100,910 | 50.9 | |
| Up to two | 84,631 | 44.3 | 2,926 | 42.7 |  | | 87,557 | 44.2 | |
| More than 2 | 44.25 | 4.8 | 459 | 6.7 |  | | 9,640 | 4.9 | |
| **Flu shot during last autumn** | 39333 | 20.6 | 2304 | 33.6 | <.001 | | 41637 | 21.0 | |
| **Anti-pneumococcal in the last 12 months** | 6822 | 3.6 | 329 | 4.8 | <.001 | | 7151 | 3.6 | |
| **Self-reported symptoms** |  |  |  |  | <.001 | |  |  | |
| **Fever** | 176432 | 92.3 | 4961 | 72.4 | <.001 | | 16714 | 8.4 | |
| **Headache** | 52400 | 27.4 | 2561 | 37.4 | <.001 | | 54961 | 27.7 | |
| **Muscle/bone pain** | 36883 | 19.3 | 2379 | 34.7 | <.001 | | 39262 | 19.8 | |
| **Olfactory and taste disorders** | 9568 | 5.0 | 1448 | 21.1 | <.001 | | 11016 | 5.6 | |
| **Shortness of breath** | 9807 | 5.1 | 1034 | 15.1 | <.001 | | 10841 | 5.5 | |
| **Chest pain** | 12191 | 6.4 | 964 | 14.1 | <.001 | | 13155 | 6.6 | |
| **Heart palpitations** | 11621 | 6.1 | 875 | 12.8 | <.001 | | 12496 | 6.3 | |
| **Gastrointestinal disturbances** | 31178 | 16.3 | 1926 | 28.1 | <.001 | | 33104 | 16.7 | |
| **Conjunctivities** | 18022 | 9.4 | 818 | 11.9 | <.001 | | 18840 | 9.5 | |
| **Sore throat/rhinorrhea** | 62,181 | 32.5 | 2529 | 36.9 | <.001 | | 64710 | 32.7 | |
| **Cough** | 39631 | 20.7 | 2367 | 34.5 | <.001 | | 41998 | 21.2 | |
| **Pneumonia** | 593 | 0.3 | 556 | 8.1 | <.001 | | 1149 | 0.6 | |
| **No symptoms** | 76712 | 40.1 | 1783 | 26.0 | <.001 | | 78495 | 39.6 | |
| **Hospitalized for COVID-19°** | 234 | 0.1 | 527 | 7.7 | <.001 | | 761 | 0.4 | |
| **Smoking status** |  |  |  |  | <.001 | |  |  | |
| Never smoked | 109,431 | 57.2 | 4,334 | 63.2 |  | | 113,765 | 57.4 | |
| Former smoker | 46,121 | 24.1 | 1,463 | 21.3 |  | | 47,584 | 24.0 | |
| Current smoker (≤10 cigarettes/day) | 19,537 | 10.2 | 599 | 8.7 |  | | 20,136 | 10.2 | |
| Current smoker (>10cigarettes/day) | 16,161 | 8.5 | 461 | 6.7 |  | | 16,622 | 8.4 | |
| Co-habitants at risk* | 36,599 | 19.1 | 1,366 | 19.9 | .11 | | 37,965 | 19.2 | |
| **Residence area** |  |  |  |  | <.001 | |  |  | |
| Countryside | 19,904 | 10.4 | 792 | 11.6 |  | | 55,224 | 27.9 | |
| Small town | 82,869 | 43.3 | 2,817 | 41.1 |  | | 36,501 | 18.4 | |
| Suburbs > 100.000 inhabitants | 35,298 | 18.5 | 1,203 | 17.5 |  | | 85,686 | 43.3 | |
| City town > 100.000 inhabitants | 53,179 | 27.8 | 2,045 | 29.8 |  | | 20,696 | 10.5 | |
| **Household crowding index#** |  |  |  |  | .01 | |  |  | |
| Low | 176,682 | 92.4 | 6,269 | 92.4 |  | | 182,951 | 92.4 | |
| Middle | 14,325 | 7.5 | 578 | 8.4 |  | | 14,903 | 7.5 | |
| High | 243 | 0.1 | 10 | 0.2 |  | | 253 | 0.1 | |
| **Contact COVID-19 cases°** | 23,387 | 12.2 | 4,861 | 70.9 | <.001 | | 28,248 | 14.3 | |
| **Weekly outing** |  |  |  |  | <.001 | |  |  | |
| Never | 33,050 | 17.3 | 1,547 | 22.6 |  | | 34,597 | 17.5 | |
| 1-3 times | 120,881 | 63.2 | 1,790 | 26.1 |  | | 122,671 | 61.9 | |
| 4 times or more | 37,319 | 19.5 | 3,520 | 51.3 |  | | 40,839 | 20.6 | |
| **Emergency number contact** |  |  |  |  | <.001 | |  |  | |
| No | 174,934 | 91.5 | 3,721 | 54.3 |  | | 178,655 | 90.2 | |
| No, but I went to a hospital on my own initiative | 214 | 0.1 | 168 | 2.5 |  | | 382 | 0.2 | |
| Yes, and they did not suggest to me self-isolation | 6,644 | 3.5 | 395 | 5.8 |  | | 7,039 | 3.4 | |
| Yes, and they suggested to me self-isolation | 9,254 | 4.8 | 2,048 | 29.9 |  | | 11,302 | 5.7 | |
| Yes, I was sent to a hospital | 204 | 0.1 | 525 | 7.7 |  | | 729 | 0.4 | |
| **Self-rate health status** |  |  |  |  | <.001 | |  |  | |
| Good | 161,191 | 84.3 | 5,511 | 80.4 |  | | 166,702 | 84.2 | |
| Adequate | 28,386 | 14.8 | 1,230 | 17.9 |  | | 29,616 | 15.0 | |
| Bad | 1,673 | 0.9 | 116 | 1.7 |  | | 1,789 | 0.9 | |

*White collar including legislators, senior officials and managers, professionals, technicians, associate professionals, clerks and service workers and shop and market sales workers: blue collar including skilled agricultural and fishery workers and craft and related trades workers, plant and machine operators and assemblers and elementary occupations, others including armed forces and unspecificed occupations.

° San Marino Republic, foreign countries or unkown.

* Elderly persons or anyone with immunocompromising or chronic disease conditions, #Number of co-habitants/number of rooms

°Suspected/confirmed

**Table S2.** **Odds ratios and relative 95%CI of positive NPS molecular test by smoking habit (N=6,857)**

|  | **All** | | **Negative** | | **Positive** | | **Model 1** | **Model 2** |
| --- | --- | --- | --- | --- | --- | --- | --- | --- |
| **Smoking habit** | **N=6,857** | | **N=5,166** | **%=75.3** | **N=1,691** | **%=24.7** | **OR (95% CI)^1^** | **aOR (95% CI)^2^** |
| ***Smoking status*** |  |  |  |  |  |  |  |  |
| Never smoked | 4,334 | 63.2 | 3210 | 62.1 | 1124 | 66.5 | 1 (ref.) | 1 (ref.) |
| Former smoker | 1,463 | 21.3 | 1056 | 20.4 | 407 | 24.1 | 0.99 (0.87-1.14) | 1.03 (0.90-1.19) |
| Current smokers | 1,060 | 15.46 | 900 | 14.4 | 160 | 9.5 | 0.54 (0.45-0.65) | 0.54 (0.44-0.65) |
| ***Dose-response relationship*** |  |  |  |  |  |  |  |  |
| Never smoked | 4,334 | 63.2 | 3210 | 62.1 | 1124 | 66.5 | 1 | 1 |
| Former smokers (≤ 10 yrs) | 645 | 9.4 | 487 | 9.4 | 158 | 9.3 | 1.00 (0.83-1.22) | 1.04 (0.85-1.27) |
| Former smokers (>10 yrs) | 818 | 11.9 | 569 | 11.0 | 249 | 14.7 | 0.98 (0.83-1.17) | 1.02 (0.86-1.22) |
| Mild smokers | 298 | 4.4 | 249 | 4.8 | 49 | 2.9 | 0.79 (0.57-1.08) | 0.76 (0.55-1.05) |
| Moderate smokers | 432 | 6.3 | 365 | 7.1 | 67 | 4.0 | 0.55 (0.42-0.72) | 0.56 (0.42-0.73) |
| Heavy smokers | 330 | 4.8 | 286 | 5.5 | 44 | 2.6 | 0.39 (0.28-0.54) | 0.38 (0.27-0.53) |
| *P-trend* |  |  |  |  |  |  | <0.001 | <0.001 |

Model 1 sex- and age-adjusted

Model 2 additionally adjusted for education, occupation, area of residence, heart diseases, lung diseases, hypertension, metabolic and oncological diseases, contact with confirmed or suspected COVID-19 cases, living area, crowding index, and living with at risk co-habitants.

Mild smokers (≤10 cigarettes/day and <15 years); Moderate smokers (≤10 cigarettes/day and ≥15 years or >10 cigarettes/day and <15 years); Heavy smokers (>10 cigarettes/day ≥15 years).

**Table S3. Adjusted odds ratios* for positive SARS-CoV-2 test by median age and smoking habit (N=6,857)**

| **Indicator variable** | **N (%)** | **aOR**  **(95% CI)** |
| --- | --- | --- |
| **Median age * smoking status** |  |  |
| Age **≤** 48 and never smokers | 2233 (32.6) | 1 (ref.) |
| Age **>** 48 and never smokers | 2101 (30.6) | 1.52 (1.31-1.76) |
| Age **≤** 48 and former smokers | 618 (9.0) | 0.98 (0.78-1.22) |
| Age **>** 48 and former smokers | 845 (12.3) | 1.66 (1.37-2.01) |
| Age **≤** 48 and current smokers | 628 (9.2) | 0.67 (0.52-0.86) |
| Age **>** 48 and current smokers | 432 (6.3) | 0.58 (0.43-0.78) |

*Adjusted for sex, education, occupation, area of residence, heart diseases, lung diseases, hypertension, metabolic and oncological diseases, contact with COVID-19 cases, living area, crowding index, and living with at risk co-habitants.

**Table S4. Adjusted odds ratios* for positive SARS-CoV-2 test by smoke-related variables (intensity, duration, and pack-years of smoking) (N=6,857)**

|  | **N=6,857** | | **Model** |
| --- | --- | --- | --- |
| **Smoking habit** | **N, %** | | **aOR (95% CI)^2^** |
| Never smoked | 4,334 | 63.2 | 1 (ref.) |
| Former smokers | 1,463 | 21.3 | 1.03 (0.90-1.19) |
| ***Intensity of smoking*** |  |  |  |
| Current smokers (≤ 10 cig/day) | 599 | 8.7 | 0.64 (0.51-0.81) |
| Current smokers (> 10 cig/day) | 461 | 6.7 | 0.42 (0.31-0.56) |
| ***Duration of smoking*** |  |  |  |
| Current smokers <15 years | 429 | 6.3 | 0.70 (0.53-0.92) |
| Current smokers 15-30 years | 462 | 6.7 | 0.49 (0.37-0.64) |
| Current smokers >30 years | 169 | 2.5 | 0.40 (0.26-0.61) |
| *P for trend* |  |  | <0.001 |
| ***Pack-years of smoking*** |  |  |  |
| 0.5-4.9 | 338 | 4.9 | 0.73 (0.54-1.00) |
| 5.0-11.2 | 348 | 5.1 | 0.51 (0.38-0.70) |
| 11.3-65.0 | 374 | 5.5 | 0.43 (0.32-0.58) |
| *P for trend* |  |  | <0.001 |
| Continuous variable |  |  | 0.83 (0.79-0.88) |

*Adjusted for age, sex, education, occupation, area of residence, heart diseases, lung diseases, hypertension, metabolic and oncological diseases, contact with COVID-19 cases, living area, crowding index, and living with at risk co-habitants.
